# Prevalence and associated factors of depressive and anxiety symptoms among healthcare workers in the post-pandemic era of COVID-19 at a tertiary hospital in Shenzhen, China: A cross-sectional study

**DOI:** 10.3389/fpubh.2023.1094776

**Published:** 2023-03-20

**Authors:** Zhiya Liang, Ying Wang, Xiaoyue Wei, Wanyi Wen, Jianping Ma, Jun Wu, Shaofen Huang, Pei Qin

**Affiliations:** ^1^Center for Clinical Epidemiology and Evidence-Based Medicine, Shenzhen Qianhai Shekou Free Trade Zone Hospital, Shenzhen, Guangdong, China; ^2^School of Public Health, Shantou University, Shantou, Guangdong, China; ^3^Department of Public Health, Shenzhen Qianhai Shekou Free Zone Hospital, Shenzhen, Guangdong, China; ^4^Department of Biostatistics and Epidemiology, School of Public Health, Shenzhen University Health Science Center, Shenzhen, Guangdong, China

**Keywords:** healthcare workers, COVID-19, depressive symptoms, anxiety symptoms, prevalence

## Abstract

**Background:**

Healthcare workers were at high risk of psychological problems during the COVID-19 pandemic, but it remains not well-investigated in the post-pandemic era of COVID-19, with regular epidemic prevention and control embedded in burdened healthcare work. This study aimed to investigate the prevalence and potential risk factors of the symptoms of depression and anxiety among healthcare workers at a tertiary hospital in Shenzhen.

**Method:**

Our cross-sectional study was conducted among 21- to 64-year-old healthcare workers in December 2021 at a tertiary hospital in Shenzhen, using a simple random sampling strategy. A wide range of socio-demographic characteristics, individual information, and psychological condition of the subjects were extracted. Healthcare workers' psychological conditions were tested with the Center for Epidemiologic Studies Depression (CESD-10), General Anxiety Disorder (GAD-7), Insomnia Severity Index (ISI), Work-Family Conflict Scale (WFCS), 10-item Connor-Davidson Resilience Scale (CD-RISC-10), and 17-item of Maslach's Burnout Inventory-Human Services Survey (MBI-HSS-17). Data were collected based on these questionnaires. Descriptive statistics were used to assess the difference between healthcare workers with depressive and anxiety symptoms among different groups. Hierarchical logistic regression analyses were conducted to investigate the association between focused variables and mental health outcomes.

**Results:**

A total of 245 healthcare workers were enrolled. The proportion of depressive symptoms, anxiety symptoms and their co-occurrence were 34.7, 59.6, and 33.1%, respectively. Logistic regression showed that for the three outcomes, no history of receiving psychological help and self-rated good or higher health were protective factors, whereas more severe insomnia and job burnout were risk factors. Junior or lower job title and higher psychological resilience were related to a lower prevalence of depressive symptoms, while relatively longer working hours and larger work-family conflict were positively associated with the anxiety symptoms. Psychological resilience was inversely associated with the co-occurrence of depressive and anxiety symptoms.

**Conclusions:**

Our study revealed a high proportion of psychological problems and proved that several similar factors which were significant during the pandemic were also associated with the symptoms of depression and anxiety among healthcare workers in the post-pandemic era of COVID-19. These results provide scientific evidence for psychological interventions for healthcare workers.

## 1. Introduction

First appearing in China's Wuhan province in December 2019 (1), coronavirus disease 2019 (COVID-19) was declared a global pandemic on March 11, 2020 (2). The WHO estimated that as of September 21, 2022, COVID-19 had infected over 609 million individuals worldwide and caused over 6.5 million fatalities (3). This pandemic has caused an unusual situation, with people worldwide suffering from various levels of fear, depression, and anxiety (4). Facing the unprecedented situation, healthcare workers have made great efforts to provide medical treatment and care to control the epidemic quickly during the COVID-19 pandemic and faced excessive workloads and psychological pressure, thereby having a large proportion of psychological problems. Compared to other occupational groups, they were particularly vulnerable to psychological problems (5–7) and more than one-third of healthcare workers experienced anxiety and depression during the COVID-19 pandemic globally (8) and in China (9).

Different from the early pandemic stage without a thorough grasp of the management of COVID-19, China and other countries around the world have already entered the post-pandemic era with routine prevention and control measures embedded in the post-pandemic new normal lifestyles (10, 11). By the time of our study, compared with the early pandemic stage, the epidemic was relatively well-controlled and a relatively strict control measures in this period were routine, which actually lasted for almost 2 years in all areas in China from the end of April 2020 (12). Due to the virus variation of COVID-19, the post-pandemic era might be prolonged for a longer time. It would profoundly impact many aspects (13), further stressing the great need to assess collateral damage in this era, such as mental health problems including depressive and anxiety symptoms (14). A study conducted in Shenzhen also proposed the concept of the post-epidemic era and investigated the prevalence and contributory factors of anxiety and depression among pregnant women during the specific period (10). Moreover, previous major public health emergencies also suggested that psychological issues may continue to exist or even develop in the post-pandemic era (13). Therefore, further investigation of the healthcare workers' psychological status in the post-epidemic era has certain scientific basis and practical significance.

Several studies have been performed during the COVID-19 pandemic to show some potential influencing factors. For example, healthcare workers aged below 40 have been found to have a higher level of depressive and anxiety symptoms than those aged above 40 (6, 15). Women have been found to be more vulnerable to the symptoms of depression (15–17) and anxiety (16, 18) during the COVID-19 pandemic. Nurses had a greater likelihood of developing depression than others, but any job type of healthcare worker developed anxiety at equal odds (6). And studies also revealed that healthcare workers with bachelor's degrees had significantly higher depression and anxiety than those with different education levels (19, 20). The intermediate job title was associated with higher anxiety and depression (5). Moreover, the level of psychological problems among the healthcare workers was reversely correlated with their years of working experience (21) and household income (22). For anxiety symptoms, being married was a risk factor (23). For depressive and anxiety symptoms, rural healthcare workers may have more psychosocial distress than urban ones during the COVID-19 pandemic (17). These results have suggested that the development of depressive and anxiety symptoms among healthcare workers during the COVID-19 pandemic is related to multiple socio-demographic factors, such as age, sex, job type, education level, job title, years of working, employment type, income, marital status, and place of residence. In the post-pandemic era, these socio-demographic factors should still be considered as important variables of the research. In addition to the basic demographic characteristics such as age and sex, the socio-economic decline caused by the mode of normal epidemic management may, to some extent, make education level, job title, income and other factors related to economy and employment become more important factors for the depressive and anxiety symptoms of healthcare workers in the post-pandemic era.

Several studies conducted during the COVID-19 pandemic showed that the alarming level of symptoms of depression and anxiety among healthcare workers could be associated with increased exposure to professional risks, unreasonable work arrangement, and individual physical and mental status (24, 25). In terms of individual-health-related factors, self-rated physical condition, chronic disease status, and psychological-help receiving state are focused factors in this field. The physical condition was closely related to the level of anxiety during the epidemic. The worse the individual's physical quality, the higher their level of depressive and anxiety symptoms (20). In addition, the presence of chronic illnesses was confirmed as a risk factor of getting emotional distress including depression and anxiety in general people (26). Previous studies have proved that psychological help for healthcare workers could reduce the emotional impact of COVID-19 (27). During the post-pandemic era of COVID-19, these effects may keep up among healthcare workers on their symptoms of depressive and anxiety. In the post-pandemic era of COVID-19, the isolation measures of normalized management might reduce the physical activity time of healthcare workers (28), which may make the influence of physical condition and chronic disease status more prominent. After the outbreak of COVID-19, under the condition of continuous optimization of psychological intervention studies among healthcare workers (29, 30), psychological-help receiving rate among healthcare workers might increase during the epidemic, so the influence of the acceptance state of psychological-help receiving state may have a downward trend in the post-epidemic era.

As for work-arrangement-related factors, researchers often focus on traditional job-related factors and specific COVID-19-related factors during the pandemic. Traditional job-related factors often include the frequency of night shifts, working hours and working intensity. The night shift arrangements (31) and working hours of more than 8 h (32) have been proven to be risk factors for depressive and anxiety symptoms among healthcare workers. The higher frequency of night shifts and longer working hours were inversely correlated with the level of these symptoms. Due to the development of COVID-19, epidemic-related factors also greatly influenced healthcare workers' emotional states. Some risk factors, such as additional workload from the sustainability of COVID-19 prevention measures in the post-pandemic period, may strongly burden healthcare workers' wellbeing (25). COVID-19-related departments such as the fever clinic, emergency departments and ICU may take on more epidemic responsibilities at work. Compared to pre-epidemic conditions, the total and regular workload may be changed because of the implementation of epidemic policy in the hospital. Few studies so far have investigated the association between COVID-19-related workload and psychological issues. In the post-pandemic era, traditional job-related factors are still important factors affecting the psychological status of healthcare workers. However, with the continuous extension of normalization management time, the influence of COVID-19-related workload on mental state may also decrease due to the increased role of positive factors such as psychological resilience.

Researchers often focus on depressive and anxiety symptoms as the main focus of psychological problems (9). However, many other psychological factors in the Chinese medical environment can affect the above two symptoms. In addition to depressive and anxiety symptoms, some psychological factors had been widely reported among healthcare workers before and during the pandemic. And previous studies have demonstrated that these psychological factors were risk factor of depressive and anxiety symptoms, which may be more affected by the COVID-19 pandemic. First, many studies showed the importance of sleep quality in mental health (33, 34). Long-standing evidence suggests that insomnia is associated with depression and anxiety (6, 35), and how this factor changes in the post-pandemic era is meaningful to find out. Second, psychological resilience, as a common mediating factor, could help individuals sustain emotional balance in the face of stress-inducing events (36). Previous studies conducted during the COVID-19 pandemic identified a protective role for psychological resilience in healthcare workers against the mental health burden (37). Third, job burnout is a feeling of anger, frustration, suspicion and paranoia at work, which is increasingly recognized among healthcare professionals (38). The feeling of job burnout is known to have a negative impact on mental health (39), which may be mitigated by psychological resilience at the same time (37). Moreover, in the COVID-19 pandemic, the misty boundary between work and personal life potentially resulted in conflicts between work and family or family and work due to changes in the workplace, home environment, and social relationships (40). For healthcare workers, the stress of work-family balance could have a detrimental effect on their performance at work, reduce their feelings of wellbeing, and even cause more serious mental illness (41–43). In the post-pandemic era, due to the constant changes in epidemic policies and work intensity, we predict that the degree of insomnia, job burnout and work-family conflict among healthcare workers would be further strengthened, and the effect of psychological resilience on the improvement of mental status will become more important.

Although previous studies showed the high appearance of mental health problems and confirmed some socio-demographic, individual-health, job-related, COVID-19-related and psychological parameters among healthcare workers during the COVID-19 pandemic, they were only based on the condition in the following months after the pandemic was announced. In the post-pandemic era of COVID-19, few studies investigated depressive and anxiety symptoms in Chinese healthcare workers. However, the current researches into past epidemics suggested that mental health problems could further develop after the pandemic's peak, with increased prevalence among vulnerable group with risk factors (13). Healthcare workers are a special group, with the similar work environment, relatively fixed posts, and the heavy medical work. If they are affected by some unavoidable factors in a short period of time during the COVID-19 pandemic, these factors are likely to continue to exist in the later period of the pandemic, and even deepen. But in the post-pandemic era of COVID-19, healthcare workers may also be disturbed by other factors that had little impact during the pandemic. This suggests that we should continue to focus on the factors that contribute to mental health problems during the pandemic, as well as continue to explore non-significant factors during the pandemic that may have an impact on psychological status in the post-pandemic. Moreover, growing evidence showed that regular screening for mental health symptoms was momentous in minimizing the psychological risk among healthcare workers (44). Therefore, there is a great need to explore its ongoing impact on healthcare workers' mental health in the post-pandemic era of COVID-19 and beyond.

This study aimed to investigate the levels of depressive and anxiety symptoms and their occurrence and explore related factors among healthcare workers during the post-pandemic era at a tertiary hospital in Shenzhen. The finding will be helpful in understanding the level of psychological symptoms among healthcare workers and provide a basis for mental health intervention targeted healthcare workers during the post-pandemic era. We hypothesize that, associated factors of depressive and anxiety symptoms among healthcare workers in the post-pandemic era are similar to those related to psychological status during the COVID-19 pandemic, but some factors may have different effects. In the post-pandemic era: (1) participants with a worse physical health condition, heavier jobs and COVID-19-related additional burden were more likely to have depressive and anxiety symptoms; (2) participants with a higher level of insomnia, more severe work-family conflict, graver job burnout and less psychological resilience were more likely to have depressive and anxiety symptoms.

## 2. Materials and methods

### 2.1. Study design and participants

A cross-sectional study was performed in December 2021 in a general tertiary hospital in Nanshan district, Shenzhen. Shenzhen Shekou Free Trade Zone Hospital is the only tertiary general hospital in Shenzhen Qianhai Shekou Free Trade Zone, with a total of 506 approved beds, covering 65 square kilometers and nearly 800,000 population. As a tertiary general hospital, it provides a full range of medical services and have the ability to provide specialized care for patients with serious or complex medical conditions. According to the Shenzhen Municipal Health Commission, there were about 140 new confirmed cases of COVID-19 in December 2021. This is a period in which the epidemic in majority area across China is under control because of the normalized epidemic prevention and control measures. A standardized electronic questionnaire was distributed. The questionnaire was designed with Wenjuanxing online platform [http://www.wjx.cn/ (China's largest professional online survey platform)], and a QR code was generated at the same time. To calculate the sample size for the study, we used the computational formula: $n={\left(\frac{Z_{1-\alpha /2}}{\delta }\right)}^{2}\times p\times \left(1-p\right),$ where n is sample size, *Z*_1−α/2_ is the z score for α, δ is the tolerance error and p is the prevalence of outcome in population. Assuming that the prevalence of depression was 27.7% (45), the required sample size at α = 0.05 and δ = 0.05 was 308. Considering a at least 20% loss to follow-up and enough sample size of multivariate regression analysis, we set the target sample size as about 400 participants in the study. A simple random sampling strategy was adopted and a total of 400 doctors and nurses were randomly selected from the list of all healthcare workers full-time working in this hospital to be recruited using computer-generated random numbers. For the participants recruitment, a well-designed electronic poster included the QR code and basic information about the project was delivered to the sampled eligible participants. After the online recruitment, 245 doctors and nurses who consented to participate in our study were enrolled in the present study and participate in the online questionnaire.

This study has been approved by the Ethics Committee of Shenzhen Qianhai Shekou Free Zone Hospital. All participants have provided online informed consent to participate in this study at the beginning of the questionnaire. Before the investigation, all participants were informed about the study background, aim, anonymous responses, confidential use of data, and voluntary participation principles.

### 2.2. Measurements

#### 2.2.1. Socio-demographic characteristics

Each participant's age, sex, job type, education level, job title, years of working, employment type, annual income, marital status, and place of residence were asked in this study.

#### 2.2.2. Individual-health-related factors

To investigate individual health conditions, three variables were collected, including the history of receiving psychological help, chronic disease, and self-rated overall physical condition. The situation of the history of receiving psychological help and chronic disease were divided into “yes” and “no.” Individuals' self-rated overall physical condition was categorized into “healthy/good” and “general or below.”

#### 2.2.3. Job-related factors

Night duty condition and weekly working hours were utilized to assess job-related factors. The night duty condition was categorized as “yes” and “no.” According to the working hours standard of healthcare workers in China, weekly worktime was divided into “under 42 h,” “42–58 h,” and “over 58 h.”

#### 2.2.4. COVID-19-related factors

The COVID-19-related factors comprised four variables, including whether working in the COVID-19-related departments or not (such as fever clinics), the change of total workload compared to pre-epidemic, the change of normal workload (non-COVID-19-related daily work) compared to pre-epidemic, and percentage of COVID-19-related work. The epidemic-related department was divided into “yes” or “no.” Options for total work compared to pre-epidemic and daily work compared to pre-epidemic included “decreased,” “steady,” and “increased.” The proportion of work related to epidemic prevention and control in the total workload was expressed as a percentage.

#### 2.2.5. Measurement of psychological factors

##### 2.2.5.1. Depression and anxiety

The validated Chinese version of the 10-item Center for Epidemiologic Studies Depression scale (CESD-10) (46) was used to measure depression. This measure was scored on a four-point Likert scale ranging from 0 (“Never”) to 3 (“Almost every day”) consisting of 10 items. The total score ranges from 0 to 30. The higher score indicated worse depression symptoms and a CESD-10 score ≥10 was indicative of depressive symptoms (47). The scale has been widely used in Chinese adults (48, 49) and has good reliability and validity (50). The Cronbach's alpha for CESD-10 was 0.74 in this study.

The validated Chinese version of the 7-item General Anxiety Disorder scale (GAD-7) was used to measure anxiety. The GAD-7 was developed by Spitzer et al. (51) and translated into Chinese by He et al. (52). It has been previously used in Chinese populations and found to be valid and reliable (53, 54). This measure was scored on a four-point Likert scale ranging from 0 (“none”), 1 (“a few days”), 2 (“more than half the days”), and 3 (“almost every day”) and consisted of seven items. The total score ranges from 0 to 21. The higher score showed worse anxiety symptoms and a GAD-7 score ≥5 was indicative of having anxiety symptoms. The Cronbach's alpha for GAD-7 in this study was 0.93.

##### 2.2.5.2. Insomnia

The severity of insomnia symptoms was assessed by the Insomnia Severity Index (ISI) (55, 56). Each item is scored from 0 (“none”/“very satisfied”) to 4 (“very severe”/“very dissatisfied”), with a total score from 0 to 28. The psychometric properties of the ISI Chinese version have been validated in Chinese populations (57, 58). Higher scores indicated more severe insomnia. In our study, the Cronbach's alpha coefficient was 0.92.

##### 2.2.5.3. Work-family conflict

The 10-item Work-Family Conflict Scale (WFCS) designed by Netemeyer et al. (59) was utilized to evaluate healthcare workers' work-family conflict. The scale included two subscales, corresponding to work-to-family conflict and family-to-work conflict, respectively. Each subscale included five items. Each of the items is scored from 1 (“totally disagree”) to 5 (“totally agree”). The Chinese version of WFCS had good reliability and validity in previous studies (60–62). Higher scores indicated a higher level of work-family conflict. The Cronbach's alpha for WFCS in the current study was 0.88.

##### 2.2.5.4. Psychological resilience

The 10-item Connor-Davidson Resilience Scale (CD-RISC-10) (46, 63) was used to measure resilience, capturing the core features of resilience over the preceding month. Items were scored on a five-point Likert scale ranging from 0 (“never”) to 4 (“always”), where higher scores represented better psychological resilience. The total score ranged from 0 to 40. This psychological resilience inventory was demonstrated to have good reliability and validity in the Chinese context (64–66). In this study, the Cronbach's alpha for CD-RISC-10 was 0.94.

##### 2.2.5.5. Job burnout

In the present study, the Chinese version of Maslach's Burnout Inventory-Human Services Survey (MBI-HSS) (67) with 17 items was used to assess the extent of job burnout of healthcare workers, revised by Zhang (68). This version of MBI-HSS-17 has been proved to have good reliability and validity when revised (68), and has been widely used in previous studies (69, 70). It comprised a total of 17 items that measure job burnout on a seven-point Likert scale, containing three dimensions: emotional exhaustion (EE, seven items), depersonalization (DA, three items), and personal accomplishment (PA, seven items). The participants were asked to rate the extent of their agreement from 0 (“totally disagree”) to 6 (“totally agree”). The dimension of personal accomplishment was reverse-scored. The rating score ranged from 0 to 102, and a higher score indicated a higher degree of job burnout. In this study, MBI-HSS-17 showed good internal consistency with a Cronbach's alpha of 0.79. Meanwhile, Cronbach's alphas for EE, DA, and PA were 0.70, 0.80, and 0.79, respectively.

### 2.3. Statistical analysis

Descriptive analyses were used for background characteristics. The results were expressed as the percentage value for categorical data and mean ± standard deviation for continuous data. To analyze Chi-square test or Fisher's exact test was used to compare the between-group difference. Hierarchical logistic regression analyses were conducted to investigate the association between main predictor variables and mental health outcomes (depressive symptoms, anxiety symptoms and their co-occurrence).

We adopted three models in the multivariate logistic regression. First, we adjusted for socio-demographic characteristics and mental health outcomes using logistic regression in model 1. Second, we additionally adjusted for individual-health-related factors, job-related factors and COVID-19-related factors in model 2. Third, to examine whether psychological factors were affected with mental health outcomes, we additionally entered all the psychological factors in model 3. The analysis procedure of model 1–3 was carried out for each of the three outcomes. The odds ratios (ORs) and 95% confidence intervals (CI) were presented. Two-sided tests with *P* < 0.05 were considered statistically significant. All data analyses were conducted using the statistical data analysis package for social sciences SPSS v.260 (IBM Corp. Released 2019. IBM SPSS Statistics for Windows, Version 26.0. Armonk, NY: IBM Corp) (71).

## 3. Results

### 3.1. Characteristics of the participants

A total of 245 participants participated in this study. The mean age of the participants was 37.5 ± 8.2 years, and most participants were aged from 30 to 50 years at the time of data collection (76.7%). More females (72.2%) participated in this survey, and 56.3% of the participants were doctors. The participants had relatively high levels of education levels, with most of the participants (93.9%) having an educational level of undergraduate or above. More than half of the participants (76%) had intermediate or higher job titles. Table 1 shows the prevalence of depressive and anxiety symptoms among healthcare workers and the characteristics of the factors studied.

**Table 1:** Characteristics of the participants in the study (*N* = 245).

**Variables**	**Total**	**Depressive symptoms**		***P*-value**	**Anxiety symptoms**		***P*-value**
		**Yes**	**No**		**Yes**	**No**	
Prevalence rates		34.7			59.6		
**Co-occurrence of depressive and anxiety symptoms 33.1%**							
**Socio-demographic characteristics**							
Age, $\bar{x}\pm s$				0.190^b^			0.381^b^
≤ 29	13.1%	25.0%	75.0%		53.1%	46.9%	
~39	56.3%	33.3%	66.7%		58.7%	41.3%	
~49	20.4%	44.0%	56.0%		66.0%	34.0%	
>50	10.2%	36.0%	64.0%		60.0%	40.0%	
Sex				0.673^b^			0.879^b^
Male	27.8%	36.8%	63.2%		58.8%	41.2%	
Female	72.2%	33.9%	66.1%		59.9%	40.1%	
Job type				0.761^b^			0.076^b^
Doctor	56.3%	35.5%	64.5%		64.5%	35.5%	
Nurse	43.7%	33.6%	66.4%		53.3%	46.7%	
Education level				0.184^b^			0.571^b^
College or below	6.1%	13.3%	86.7%		46.7%	53.3%	
Undergraduate	69.8%	36.8%	63.2%		60.2%	39.8%	
Postgraduate or above	24.1%	33.9%	66.1%		61.0%	39.0%	
Job title				**0.028** ^b^			**0.020** ^b^
Junior or lower	24.0%	20.3%	79.7%		44.1%	55.9%	
Intermediate	58.0%	38.7%	61.3%		64.1%	35.9%	
Senior	18.0%	40.9%	59.1%		65.9%	34.1%	
Years of working				0.233^b^			0.609^b^
≤ 10	43.7%	29.0%	71.0%		56.1%	43.9%	
~20	34.3%	40.5%	59.5%		61.9%	38.1%	
>20	22.0%	37.0%	63.0%		63.0%	37.0%	
Annual income				0.410^b^			0.143^b^
<200,000	33.1%	32.1%	67.9%		51.9%	48.1%	
~300,000	36.7%	40.0%	60.0%		66.7%	33.3%	
>300,000	30.2%	31.1%	68.9%		59.5%	40.5%	
Marital status				0.142^b^			0.185^b^
Unmarried	19.2%	25.5%	74.5%		51.1%	48.9%	
Married^a^	80.8%	36.9%	63.1%		61.6%	38.4%	
Place of residence				0.760^b^			0.174^b^
Urban	87.3%	35.0%	65.0%		61.2%	38.8%	
Rural	12.7%	32.3%	67.7%		48.4%	51.6%	
**Individual-health-related factors**							
History of receiving psychological help				**0.027** ^b^			0.130^d^
Yes	4.9%	66.7%	33.3%		83.3%	16.7%	
No	95.1%	33.0%	67.0%		58.4%	41.6%	
Chronic disease				0.289^b^			0.656^b^
Yes	12.2%	43.3%	56.7%		63.3%	36.7%	
No	87.8%	33.5%	66.5%		59.1%	40.9%	
Self-rated overall physical condition				**0.001** ^b^			**0.011** ^b^
Healthy/good	84.9%	30.3%	69.7%		56.3%	43.7%	
General or below	15.1%	59.5%	40.5%		78.4%	21.6%	
**Job-related factors**							
Night duty				0.641^b^			0.777^b^
Yes	74.7%	35.5%	64.5%		60.1%	39.9%	
No	25.3%	32.3%	67.7%		58.1%	41.9%	
Weekly working hours				0.226^b^			**0.002** ^b^
≤ 42	47.3%	31.9%	68.1%		50.9%	49.1%	
~58	35.9%	33.0%	67.0%		60.2%	39.8%	
>58	16.7%	46.3%	53.7%		82.9%	17.1%	
**COVID-19-related factors**							
COVID-19-related departments				0.768^b^			0.944^b^
Yes	22.4%	36.4%	63.6%		60.0%	40.0%	
No	77.6%	34.2%	65.8%		59.5%	40.5%	
Total workload compared to pre-epidemic				0.473^b^			**0.003** ^b^
Decreased	15.9%	38.5%	61.5%		69.2%	30.8%	
Steady	18.4%	26.7%	73.3%		37.8%	62.2%	
Increased	65.7%	36.0%	64.0%		63.4%	36.6%	
Normal workload compared to pre-epidemic				0.481^b^			**0.030** ^b^
Decreased	15.5%	36.8%	63.2%		68.4%	31.6%	
Steady	22.0%	27.8%	72.2%		44.4%	55.6%	
Increased	62.4%	36.6%	63.4%		62.7%	37.3%	
Percentage of COVID-19-related work	40.6 ± 22.6	42.2 ± 21.7	39.7 ± 23.1	0.409^b^	41.3 ± 22.8	39.6 ± 22.5	0.577^b^
**Psychological factors**							
ISI (score range: 0–28)	8.2 ± 5.9	12.8 ± 5.3	5.7 ± 4.7	**<0.001** ^c^	10.3 ± 5.8	5.1 ± 4.6	**<0.001** ^c^
WFCS (score range: 10–50)	27.3 ± 6.4	31.0 ± 5.2	25.4 ± 6.0	**<0.001** ^c^	29.7 ± 5.4	23.9 ± 6.1	**<0.001** ^c^
CD-RISC-10 (score range: 0–40)	26.1 ± 6.1	22.3 ± 5.7	28.2 ± 5.4	**<0.001** ^c^	24.0 ± 5.6	29.2 ± 5.6	**<0.001** ^c^
MBI-HSS-17 (score range: 0–102)	35.1 ± 14.1	46.1 ± 10.6	29.3 ± 12.2	**<0.001** ^c^	41.3 ± 11.7	26.0 ± 12.4	**<0.001** ^c^

N, number; ISI, Insomnia Severity Index; WFCS, Work-Family Conflict Scale; CD-RISC-10, 10-item Connor-Davidson Resilience Scale; MBI-HSS-17, 17-item Maslach's Burnout Inventory-Human Services Survey.

Data are shown as means ± standard deviation, median (interquartile range), or numbers (percentages).

Values in bold indicate statistical significance at P <0.05, and or unless otherwise noted.

a Married category included widowed and divorced participants.

b Chi-square test was used.

c Student's t-test was used.

d Fisher's exact test was used.

### 3.2. Prevalence and factors associated with depressive symptoms among healthcare workers

In this study, 34.7% of healthcare workers experienced depression symptoms. Table 2 shows the results of hierarchical logistic regressions for depressive symptoms. First, model 1 assessed the relationship between socio-demographic characteristics and depressive symptoms. Junior or lower job title (OR = 0.199, 95% CI 0.044–0.899) was found to be associated with lower odds in depressive symptoms. After adjustment for all socio-demographic variables, model 2 showed that no history of receiving psychological help (OR = 0.179, 95% CI 0.040–0.793) and healthier self-rated overall physical condition (OR = 0.289, 95% CI 0.116–0.717) had decreased odds of depressive symptoms. In model 3, a higher score of ISI (OR = 1.311, 95% CI 1.165–1.475) and MBI-HSS-17 (OR = 1.108, 95% CI 1.045–1.175) had a positive association with depressive symptoms, whereas a higher score of CD-RISC-10 (OR = 0.837, 95% CI 0.751–0.934) had a negative association with depressive symptoms.

**Table 2:** Logistic regression analysis of depressive symptoms in the participants.

**Depressive symptoms**	**Model 1**		**Model 2**		**Model 3**	
	**OR (95%** ***CI*****)**	***P*** **-value**	**OR (95%** ***CI*****)**	***P*** **-value**	**OR (95%** ***CI*****)**	***P*** **-value**
**Socio-demographic characteristics**						
Age	1.203 (0.603–2.402)	0.600	1.220 (0.575–2.589)	0.604	1.773 (0.524–6.004)	0.357
**Sex**						
Male		Ref.		Ref.		Ref.
Female	0.822 (0.422–1.598)	0.562	0.601 (0.266–1.357)	0.220	0.581 (0.151–2.240)	0.430
**Job type**						
Doctor		Ref.		Ref.		Ref.
Nurse	1.334 (0.653–2.725)	0.428	1.787 (0.790–4.042)	0.163	2.516 (0.662–9.568)	0.176
**Education level**						
Postgraduate or above		Ref.		Ref.		Ref.
Undergraduate	0.835 (0.376–1.854)	0.657	0.869 (0.362–2.088)	0.754	0.909 (0.226–3.666)	0.894
College or below	0.309 (0.052–1.846)	0.198	0.277 (0.040–1.904)	0.192	0.088 (0.004–1.889)	0.120
**Job title**						
Senior		Ref.		Ref.		Ref.
Intermediate	0.581 (0.210–1.606)	0.296	0.441 (0.135–1.446)	0.177	0.419 (0.067–2.630)	0.353
Junior or lower	0.199 (0.044–0.899)	**0.036**	0.168 (0.032–0.889)	**0.036**	0.253 (0.017–3.717)	0.316
**Years of working**						
>20		Ref.		Ref.		Ref.
~20	1.549 (0.534–4.495)	0.420	1.473 (0.468–4.638)	0.508	2.704 (0.383–19.069)	0.318
≤ 10	1.353 (0.360–5.084)	0.654	1.693 (0.396–7.242)	0.478	1.186 (0.107–13.202)	0.889
**Annual income**						
< 200,000		Ref.		Ref.		Ref.
~300,000	1.093 (0.541–2.207)	0.804	0.347 (0.122–0.986)	**0.047**	0.941 (0.281–3.151)	0.921
>300,000	0.453 (0.186–1.107)	0.083	1.030 (0.481–2.208)	0.939	0.836 (0.170–4.098)	0.825
**Marital status**						
Unmarried		Ref.		Ref.		Ref.
Married^a^	1.209 (0.520–2.810)	0.659	1.571 (0.629–3.929)	0.334	1.342 (0.311–5.786)	0.694
**Place of residence**						
Rural		Ref.		Ref.		Ref.
Urban	0.571 (0.208–1.567)	0.276	0.614 (0.205–1.838)	0.383	0.255 (0.042–1.545)	0.137
**Individual-health-related factors**						
**History of receiving psychological help**						
Yes				Ref.		Ref.
No			0.179 (0.040–0.793)	**0.023**	0.092 (0.004–1.903)	0.123
**Chronic disease**						
Yes				Ref.		Ref.
No			1.272 (0.480–3.372)	0.628	1.594 (0.378–6.722)	0.526
**Self-rated overall physical condition**						
General or below				Ref.		Ref.
Healthy/good			0.289 (0.116–0.717)	**0.007**	0.738 (0.190–2.857))	0.660
**Job-related factors**						
**Night duty**						
Yes				Ref.		Ref.
No			0.727 (0.316–1.675)	0.454	1.795 (0.485–6.640)	0.381
**Weekly working hours**						
≤ 42				Ref.		Ref.
~58			1.718 (0.708–4.164)	0.231	1.267 (0.312–5.145)	0.741
>58			1.126 (0.557–2.277)	0.741	1.489 (0.498–4.451)	0.476
**COVID-19-related factors**						
**COVID-19-related departments**						
Yes				Ref.		Ref.
No			0.899 (0.408–1.981)	0.791	3.161 (0.860–11.621)	0.083
**Total workload compared to pre-epidemic**						
Decreased				Ref.		Ref.
Steady			0.723 (0.193–2.704)	0.630	0.645 (0.077–5.420)	0.686
Increased			1.088 (0.294–4.035)	0.899	0.935 (0.132–6.599)	0.946
**Normal workload compared to pre-epidemic**						
Decreased				Ref.		Ref.
Steady			0.891 (0.254–3.131)	0.857	1.268 (0.171–9.416)	0.816
Increased			1.212 (0.324–4.537)	0.776	0.985 (0.134–7.246)	0.988
Percentage of COVID-19-related work			0.999 (0.985–1.013)	0.864	0.983 (0.960–1.007)	0.164
**Psychological factors**						
ISI total scores					1.311 (1.165–1.475)	**<0.001**
WFCS total scores					1.088 (0.979–1.210)	0.117
CD-RISC-10 total scores					0.837 (0.751–0.934)	**0.001**
MBI-HSS-17 total scores					1.108 (1.045–1.175)	**0.001**

a Married category included widowed and divorced participants.

OR, Odds Ratios; CI, Confidence Interval; ISI, Insomnia Severity Index; WFCS, Work-Family Conflict Scale; CD-RISC-10, 10-item Connor-Davidson Resilience Scale; MBI-HSS-17, 17-item Maslach's Burnout Inventory-Human Services Survey.

Odds ratios (OR) and 95% confidence intervals (CI) were derived from the multivariate logistic regression model.

Values in bold indicate statistical significance at P < 0.05.

### 3.3. Prevalence and factors associated with anxiety symptoms among healthcare workers

The prevalence of anxiety symptoms was 59.6% in this study. Table 3 shows the results of hierarchical logistic regressions for anxiety symptoms. In model 2, after adjustment for all socio-demographic variables, healthier self-rated overall physical condition (OR = 0.310, 95% CI 0.112–0.858) had decreased odds of anxiety symptoms, whereas 42–58 weekly working hours (OR = 4.507, 95% CI 1.629–12.473) had increased odds of anxiety symptoms. Model 3 found that a higher score of ISI (OR = 1.156, 95% CI 1.057–1.264), WFCS (OR = 1.103, 95% CI 1.012–1.212), and MBI-HSS-17 (OR = 1.070, 95% CI 1.022–1.121) had a positive association with depressive symptoms.

**Table 3:** Logistic regression analysis of anxiety symptoms in the participants.

**Anxiety symptoms**	**Model 1**		**Model 2**		**Model 3**	
	**OR (95%** ***CI*****)**	***P*** **-value**	**OR (95%** ***CI*****)**	***P*** **-value**	**OR (95%** ***CI*****)**	***P*** **-value**
**Socio-demographic characteristics**						
Age	0.663 (0.343–1.283)	0.223	0.571 (0.273–1.195)	0.137	0.430 (0.162–1.140)	0.090
**Sex**						
Male		Ref.		Ref.		Ref.
Female	1.140 (0.596–2.183)	0.692	0.968 (0.433–2.161)	0.937	1.042 (0.353–3.081)	0.940
**Job type**						
Doctor		Ref.		Ref.		Ref.
Nurse	0.621 (0.308–1.251)	0.182	0.819 (0.365–1.835)	0.627	0.437 (0.153–1.250)	0.123
**Education level**						
Postgraduate or above		Ref.		Ref.		Ref.
Undergraduate	1.226 (0.562–2.673)	0.609	1.400 (0.590–3.322)	0.445	1.489 (0.444–4.991)	0.519
College or below	1.241 (0.323–4.775)	0.753	1.219 (0.281–5.287)	0.792	1.264 (0.187–8.540)	0.810
**Job title**						
Senior		Ref.		Ref.		Ref.
Intermediate	0.630 (0.229–1.729)	0.369	0.533 (0.163–1.747)	0.299	0.614 (0.124–3.034)	0.549
Junior or lower	0.265 (0.064–1.107)	0.069	0.220 (0.043–1.127)	0.069	0.717 (0.079–6.482)	0.767
**Years of working**						
>20		Ref.		Ref.		Ref.
~20	0.800 (0.279–2.290)	0.678	0.630 (0.199–1.995)	0.433	0.241 (0.050–1.160)	0.076
≤ 10	0.759 (0.208–2.776)	0.677	0.749 (0.176–3.183)	0.695	0.160 (0.022–1.158)	0.070
**Annual income**						
< 200,000		Ref.		Ref.		Ref.
~300,000	1.457 (0.740–2.869)	0.276	1.376 (0.651–2.906)	0.403	1.779 (0.665–4.762)	0.251
>300,000	0.817 (0.352–1.895)	0.638	0.559 (0.206–1.517)	0.254	0.837 (0.232–3.024)	0.786
**Marital status**						
Unmarried		Ref.		Ref.		Ref.
Married^a^	1.325 (0.612–2.871)	0.476	1.696 (0.725–3.965)	0.223	1.739 (0.575–5.264)	0.327
**Place of residence**						
Rural		Ref.		Ref.		Ref.
Urban	1.070 (0.435–2.631)	0.883	0.967 (0.357–2.622)	0.948	0.989 (0.262–3.738)	0.987
**Individual-health-related factors**						
**History of receiving psychological help**						
Yes				Ref.		Ref.
No			0.288 (0.048–1.720)	0.172	0.122 (0.006–2.323)	0.162
**Chronic disease**						
Yes				Ref.		Ref.
No			1.570 (0.589–4.186)	0.367	1.568 (0.446–5.511)	0.483
**Self-rated overall physical condition**						
General or below				Ref.		Ref.
Healthy/good			0.310 (0.112–0.858)	**0.024**	0.763 (0.201–2.888)	0.690
**Job-related factors**						
**Night duty**						
Yes				Ref.		Ref.
No			0.989 (0.441–2.220)	0.980	1.189 (0.412–3.435)	0.749
**Weekly working hours**						
≤ 42				Ref.		Ref.
~58			4.507 (1.629–12.473)	**0.004**	6.602 (1.675–26.013)	**0.007**
>58			1.588 (0.820–3.076)	0.170	2.078 (0.849–5.085)	0.109
**COVID-19-related factors**						
**COVID-19-related departments**						
Yes				Ref.		Ref.
No			0.918 (0.420–2.006)	0.830	2.242 (0.804–6.255)	0.123
**Total workload compared to pre-epidemic**						
Decreased				Ref.		Ref.
Steady			0.364 (0.096–1.381)	0.137	0.329 (0.048–2.236)	0.255
Increased			0.873 (0.225–3.393)	0.845	1.102 (0.174–6.995)	0.918
**Normal workload compared to pre-epidemic**						
Decreased				Ref.		Ref.
Steady			0.734 (0.204–2.641)	0.636	0.765 (0.125–4.672)	0.771
Increased			0.933 (0.245–3.558)	0.919	0.584 (0.098–3.497)	0.556
Percentage of COVID-19-related work			0.997 (0.983–1.012)	0.709	0.988 (0.969–1.008)	0.238
**Psychological factors**						
ISI total scores					1.156 (1.057–1.264)	**0.001**
WFCS total scores					1.103 (1.012–1.202)	**0.026**
CD-RISC-10 total scores					0.919 (0.839–1.006)	0.068
MBI-HSS-17 total scores					1.070 (1.022–1.121)	**0.004**

a Married category included widowed and divorced participants.

OR, Odds Ratios; CI, Confidence Interval; ISI, Insomnia Severity Index; WFCS, Work-Family Conflict Scale; CD-RISC-10, 10-item Connor-Davidson Resilience Scale; MBI-HSS-17, 17-item Maslach's Burnout Inventory-Human Services Survey.

Odds ratios (OR) and 95% confidence intervals (CI) were derived from the multivariate logistic regression model.

Values in bold indicate statistical significance at P < 0.05.

### 3.4. Co-occurrence and factors associated with the symptoms of depression and anxiety among healthcare workers

The co-occurrence of the symptoms of depression and anxiety among healthcare workers was 33.1% in this study. Table 4 shows the results of hierarchical logistic regressions for co-occurrence of depressive and anxiety symptoms. After adjustment for all socio-demographic variables, Model 2 showed that no history of psychological help (OR = 0.162, 95% CI 0.036–0.736) and healthier self-rated overall physical condition (OR = 0.251, 95% CI 0.099–0.636) had decreased odds of the co-occurrence. Model 3 found that a higher score of ISI (OR = 1.285, 95% CI 1.146–1.441) and MBI-HSS-17 (OR = 1.126, 95% CI 1.058–1.199) had a positive association with the co-occurrence, whereas a higher score of CD-RISC-10 (OR = 0.842, 95% CI 0.757–0.937) had a negative association with the co-occurrence.

**Table 4:** Logistic regression analysis of the co-occurrence of the symptoms of depression and anxiety in the participants.

**Co-occurrence**	**Model 1**		**Model 2**		**Model 3**	
	**OR (95%** ***CI*****)**	***P*** **-value**	**OR (95%** ***CI*****)**	***P*** **-value**	**OR (95%** ***CI*****)**	***P*** **-value**
**Socio-demographic characteristics**						
Age	1.123 (0.557–2.265)	0.745	1.138 (0.527–2.459)	0.741	1.448 (0.398–5.269)	0.575
**Sex**						
Male		Ref.		Ref.		Ref.
Female	0.790 (0.405–1.542)	0.490	0.511 (0.222–1.177)	0.115	0.388 (0.096–1.564)	0.183
**Job type**						
Doctor		Ref.		Ref.		Ref.
Nurse	1.051 (0.511–2.162)	0.893	1.422 (0.620–3.261)	0.406	1.326 (0.331–5.307)	0.690
**Education level**						
Postgraduate or above		Ref.		Ref.		Ref.
Undergraduate	0.774 (0.346–1.729)	0.532	0.795 (0.324–1.951)	0.617	0.882 (0.210–3.707)	0.864
College or below	0.326 (0.054–1.948)	0.219	0.306 (0.044–2.111)	0.230	0.116 (0.006–2.364)	0.162
**Job title**						
Senior		Ref.		Ref.		Ref.
Intermediate	0.620 (0.221–1.736)	0.363	0.419 (0.124–1.423)	0.163	0.496 (0.070–3.491)	0.481
Junior or lower	0.242 (0.053–1.102)	0.067	0.194 (0.036–1.055)	0.058	0.543 (0.035–8.548)	0.664
**Years of working**						
>20		Ref.		Ref.		Ref.
~20	1.126 (0.295–4.297)	0.862	1.300 (0.399–4.234)	0.663	0.631 (0.123–3.250)	0.582
≤ 10	1.376 (0.468–4.045)	0.561	1.436 (0.323–6.378)	0.634	0.863 (0.245–3.042)	0.819
**Annual income**						
< 200,000		Ref.		Ref.		Ref.
~300,000	1.090 (0.537–2.210)	0.812	1.024 (0.472–2.221)	0.952	0.863 (0.245–3.042)	0.819
>300,000	0.401 (0.161–0.999)	0.050	0.292 (0.099–0.860)	**0.025**	0.631 (0.123–3.250)	0.582
**Marital status**						
Unmarried		Ref.		Ref.		Ref.
Married^a^	1.385 (0.587–3.269)	0.457	1.807 (0.706–4.625)	0.217	1.991 (0.424–9.349)	0.383
**Place of residence**						
Rural		Ref.		Ref.		Ref.
Urban	0.552 (0.201–1.518)	0.249	0.616 (0.204–1.858)	0.390	0.237 (0.038–1.474)	0.123
**Individual-health-related factors**						
**History of receiving psychological help**						
Yes				Ref.		Ref.
No			0.162 (0.036–0.736)	**0.018**	0.063 (0.003–1.574)	0.092
**Chronic disease**						
Yes				Ref.		Ref.
No			1.126 (0.416–3.044)	0.816	1.133 (0.241–5.325)	0.874
**Self-rated overall physical condition**						
General or below				Ref.		Ref.
Healthy/good			0.251 (0.099–0.636)	**0.004**	0.533 (0.132–2.150)	0.377
**Job-related factors**						
**Night duty**						
Yes				Ref.		Ref.
No			0.602 (0.253–1.432)	0.251	1.459 (0.373–5.704)	0.587
**Weekly working hours**						
≤ 42				Ref.		Ref.
~58			1.907 (0.776–4.684)	0.159	1.331 (0.301–5.879)	0.706
>58			1.170 (0.568–2.407)	0.671	1.463 (0.460–4.654)	0.519
**COVID-19-related factors**						
**COVID-19-related departments**						
Yes				Ref.		Ref.
No			1.022 (0.455–2.294)	0.958	4.581 (1.151–18.225)	**0.031**
**Total workload compared to pre-epidemic**						
Decreased				Ref.		Ref.
Steady			0.845 (0.221–3.233)	0.806	1.072 (0.120–9.596)	0.950
Increased			1.178 (0.305–4.555)	0.812	1.328 (0.189–9.331)	0.775
**Normal workload compared to pre-epidemic**						
Decreased				Ref.		Ref.
Steady			0.927 (0.259–3.320)	0.907	1.458 (0.170–12.505)	0.731
Increased			1.274 (0.327–4.962)	0.727	1.056 (0.131–8.492)	0.959
Percentage of COVID-19-related work			1.000 (0.985–1.015)	0.988	0.985 (0.960–1.010)	0.238
**Psychological factors**						
ISI total scores					1.285 (1.146–1.441)	**<0.001**
WFCS total scores					1.097 (0.983–1.224)	0.097
CD-RISC-10 total scores					0.842 (0.757–0.937)	**0.002**
MBI-HSS-17 total scores					1.126 (1.058–1.199)	**<0.001**

a Married category included widowed and divorced participants.

OR, Odds Ratios; CI, Confidence Interval; ISI, Insomnia Severity Index; WFCS, Work-Family Conflict Scale; CD-RISC-10, 10-item Connor-Davidson Resilience Scale; MBI-HSS-17, 17-item Maslach's Burnout Inventory-Human Services Survey.

Odds ratios (OR) and 95% confidence intervals (CI) were derived from the multivariate logistic regression model.

Values in bold indicate statistical significance at P < 0.05.

## 4. Discussion

This study examined the prevalence of depressive symptoms, anxiety symptoms, and their co-occurrence among Chinese healthcare workers during the post-pandemic era and to identify key socio-demographic, job-related and psychological correlates of these disorders. We found a prevalence of 34.7% for depressive symptoms, 59.6% for anxiety symptoms, and 33.1% for the co-occurrence of depressive and anxiety symptoms. Our results showed that for depressive symptoms, anxiety symptoms and their co-occurrence, no history of receiving psychological help and self-rated good or higher health were protective factors, whereas more severe insomnia and job burnout were risk factors. Junior or lower job title and a higher score of psychological resilience were related to a lower prevalence of depressive symptoms, while relatively longer working hours and larger work-family conflict were positively associated with the anxiety symptoms. At the same time, a higher score of psychological resilience was inversely associated with co-occurrence of depressive and anxiety symptoms.

Lasting almost for 3 years, healthcare workers have been playing a major role in fighting against COVID-19 and might suffer from a large burden of psychological problems. Understanding their level of depressive/anxiety symptoms and associated factors can help hospital management and government policymakers to take effective measures to enhance the mental health status of healthcare workers, thus improving their professional performance and work efficiency. Our study conducted a cross-sectional survey on mental health status among healthcare workers in the post-pandemic era of COVID-19, about the third year since the first wave. The prevalence rates of depressive and anxiety symptoms for healthcare workers in the post-pandemic era were generally comparable to those observed in previous studies during the COVID-19 pandemic era, but different from the prevalence before the pandemic. In this study, we found that the prevalence of depressive symptoms was 34.7%, which was nearly in line with that reported by Deng et al. in a meta-analysis (72) (31%) and Tong et al. (7) (37%) among healthcare workers in the early stages of the COVID-19 pandemic in China. Compared to Lai et al.'s study (5) (50.4%) among healthcare workers at the peak of the pandemic in China, our study found a lower prevalence in the post-pandemic era, which may be because of the potential decreased fear and uncertainty from a better understanding of the COVID-19 and some appropriate interventions taken to relieve depressive symptoms during the pandemic. The prevalence of depressive symptoms was almost consistent with that reported by Liang et al. (73) (39.5%) in the late stage of the COVID-19 pandemic, which proved that the prevalence of depressive symptoms remains relatively high level in the post-pandemic. For anxiety symptoms, our study found a higher prevalence than the meta-analysis [40% reported by Deng et al. (72)] and previous studies among healthcare workers in China reported by Li et al. (31) (20.6%) and Lai et al. (5) (44.6%) during the COVID-19 pandemic era. Moreover, the prevalence of anxiety symptoms was higher than that reported by Liang et al. (73) (26.0%) in the late stage of the COVID-19 pandemic. The reason for the higher prevalence in our study may result from different pressure from life and work and different prevention and control measures for healthcare workers living in different areas. Our findings first showed that there were as high as 33.1% of healthcare workers suffering from the co-occurrence of depressive and anxiety symptoms, which was higher than that in Cameroon (14.73%) during the COVID-19 pandemic era (74). Moreover, consistent with our results, previous studies have reported an increase in healthcare workers' levels of depressive and anxiety symptoms as the COVID-19 outbreak continued (25). A study on Southeast Asian adults (75) also showed a higher prevalence of psychological problems after 18 months of the declaration of the pandemic compared to the 1st year. In the present study, we are the first study to report the high prevalence of depressive and anxiety symptoms as well as the co-occurrence among healthcare workers at a tertiary hospital in Shenzhen, a city with a large transient population, suggesting a long-lasting psychological impact of the pandemic among healthcare workers in the future. Given the widespread psychological crisis among healthcare workers in the post-pandemic era of COVID-19, it is necessary to provide various support to promote their psychological health in the post-pandemic era.

The findings of our study found few socio-demographic characteristics associated with the prevalence of depressive and anxiety symptoms among healthcare workers in the post-pandemic era. In this study, a junior or lower job title was a protective factor for the development of depressive symptoms in healthcare workers in the post-pandemic era of COVID-19. However, the results regarding job titles were inconsistent with previous studies during the COVID-19 pandemic in China. A meta-analysis during the COVID-19 epidemic has proved that healthcare workers with lower job titles have higher prevalence rates of depressive symptoms than those with higher job titles (76). A cross-sectional study in Anhui Province has also showed that the depression level of healthcare workers with middle and junior job titles were higher than those with senior titles during the pandemic (77). This difference might be due to the different demands for healthcare workers with different job titles at different stages of the epidemic. During the COVID-19 pandemic, due to the continuous outbreaks of the epidemic, a large number of healthcare workers with low job titles were assigned to perform the large-scale nucleic acid testing and epidemic-related prevention and control work. Therefore, the influence of lower job title on the level of depressive symptoms was more obvious. However, in the post-pandemic era, with the maturity of the overall level of epidemic prevention and control, the demand for healthcare workers with lower job titles was not as great as that during the epidemic. The difficulties in medical work have gradually returned to positions with higher job titles requiring higher level of medical technology. Therefore, lower job titles replaced the place of risk factor during the pandemic as a protective factor for depressive symptoms among healthcare workers in the post-epidemic era. Our results suggest that in the post-pandemic era, more attention should be paid to depressive symptoms among healthcare workers with high job titles in the post-pandemic era. However, further research is required to investigate the association between job titles and depressive symptoms, and individualized interventions might be more useful for healthcare workers in different regions of our country.

Although chronic disease condition, night duty condition and COVID-19-related factors, including whether working in a COVID-19-related department or not, the change of the COVID-19-related total/normal workload compared to pre-epidemic, or the percentage of COVID-19-related work were not statistically significant in our logistic regression models, our results showed that the level of anxiety symptoms for people with longer weekly working hours (42–58 h) was significantly higher than shorter weekly working hours ( ≤ 42 h). Increased working hours were one of the important factors contributing to developing anxiety symptoms, and similar findings have been reported in studies during the pandemic (18, 32). Our study showed that the increase in working hours was still an important factor affecting psychological status, with a greater effect on anxiety symptoms among traditional job-related factors in the post-pandemic era. Besides, no history of psychological help was shown to be protective factors for depressive and the co-occurrence in the post-pandemic era, which was inconsistent with previous study (27). The reason may be that the effect of psychological help is diminishing as the epidemic continues to develop. During the epidemic, timely psychological help had a good effect on the relief of healthcare workers who just suffered from stress brought by the epidemic, so the popularity rate of psychological help has been greatly improved. Therefore, in the post-pandemic era, people who have not received psychological help may be those with milder psychological problems. The result inspires us that in order to improve the effect of psychological help on psychological problems in the post-epidemic era, it is necessary to reform the content, form and scope of psychological help. Moreover, good or higher self-rated overall physical condition was inversely associated with depressive symptoms, anxiety symptoms and their co-occurrence in the post-pandemic era, same as the case in previous study during the pandemic (20). Our result suggests that, as during the pandemic, physical condition remains a very important factor in psychological problems in the post-pandemic era. The main reason for the non-significant association of COVID-19-related factors may be that the role of COVID-19 related work effects may have become smaller, or that our survey time was too short to cover various stages of the pandemic to better indicate the impact of work factors. In the future, the impact of COVID-19-related factors on the mental health of healthcare workers in the post-pandemic era needs further research.

Consistent with previous studies (20, 35) during the pandemic, healthcare workers with insomnia were found more likely to have depressive and anxiety symptoms than those with good sleep quality in the post-pandemic era. A study conducted in Bangladesh (35) reported that physicians who were suffering from sleep problems had a higher level of depressive symptoms compared to those who had never suffered from sleep problems. Cheng et al. also demonstrated a moderate association between the level of anxiety and sleep quality among pediatric healthcare workers (20). In fact, several studies have shown a link between insomnia and mental conditions including depression and anxiety during the COVID-19 pandemic in different countries (78–80). The finding suggests that insomnia was a relatively long-lasting factors both during the pandemic and post-pandemic era. Continuous measures should be taken to enhance their sleep quality to improve the mental health status of healthcare workers.

We found that people with a higher level of psychological resilience had a lower level of depressive symptoms and co-occurrence in the post-pandemic era, which agreed with previous studies during the COVID-19 pandemic (39, 80). The finding might be explained by the fact that healthcare workers with different levels of resilience could moderate the effects of perceived stress on depression by adopting different coping styles (81). In the post-pandemic era, our study found that work-family conflict was still be positively associated with anxiety symptoms while job burnout was also found to be positively associated with depressive symptoms, anxiety symptoms and their co-occurrence among healthcare workers, which were also confirmed in other studies during the pandemic (41, 42, 82–84). Lv et al. stated that healthcare workers with high levels of work-family conflict are particularly vulnerable to mental health problems (41). A study in Brazil also reported a positive relationship between burnout and depressive symptoms among workers at a university hospital (19). On the one hand, the workload and working hours of healthcare workers would fluctuate as the pandemic evolved, which was inevitable to bring negative effects to their family life (85) such as increased work-family conflict and thereby increased the level of anxiety symptoms (86). On the other hand, in the post-pandemic era of COVID-19, the uncertainty and variable conditions related to the pandemic can lead to role pressure for their families (87) and in the hospital and aggravate the severity of their work-family conflict (88). Healthcare workers' job burnout will not only reduce work efficiency and cause medical malpractices (89), but also increase the risk for depressive symptoms (90). In the hospital workplace, many healthcare workers might become burnout due to work-family conflict and the doctor-patient relationship in the post-pandemic era, being unable to cope with negative emotions and thus falling into a depressive and anxious state (91). We proved that psychological factors are important for depressive and anxiety symptoms both in the pandemic and the post-pandemic era. However, our study also showed that in the post-pandemic era, work-family conflict mainly positively affected anxiety symptoms, while job burnout positively affected depressive symptoms, anxiety symptoms, and their co-occurrence. Our results suggest that actions can be go on to alleviate work-family conflict and job burnout to decrease mental health problems and enhance positive cognition in healthcare workers in the post-pandemic era (88). Moreover, in designing psychological intervention strategies during the post-epidemic era, more consideration can be given to starting from job burnout, which may have better effects and solve psychological problems more efficiently.

Our study was the first to investigate the mental health problems in the post-pandemic era in China, especially in the special period with under-control pandemic due to very strict routine prevention and control measures. Furthermore, to our knowledge, no study has explored the factors associated with mental health problems among healthcare workers in this special period and we have investigated a variety of factors of depressive and anxiety symptoms and their co-occurrence. Our study indicates that the mental health among healthcare workers in the post-pandemic era needs more attention, and timely screening and early intervention are needed. The results also indicate that there are some common factors during the pandemic and the post-pandemic era. It is reasonable that some factors such as poor sleep quality, work-family conflict, and job burnout in healthcare workers that pose a threat to their mental health during the COVID-19 pandemic may continue to exist in the post-pandemic era. But with the dynamic change in individual psychological status such as worrying about being infected and other factors such as workload under different social background, we considered that the effect of these factors may be different in the development of mental health problems at the two different stages. For example, in our study, we found that higher job titles have negative effect on mental health in post-pandemic era, which was different from the findings during the pandemic (76, 77). The finding might be explained by the higher psychological stress and burnout due to very long duration to cope with different tasks from the regular prevention of COVID-19 pandemic. Therefore, it would be interesting for researchers to explore the difference in the effect of such factors on depressive and anxiety symptoms between the two periods. Our study also had some important implications. In our study, lower job titles and no history of receiving psychological help are protective factors in the post-epidemic era, which suggested that healthcare authorities and policymakers should focus more on healthcare workers with higher job titles and longer working hours. Compared to other types of healthcare workers, regular psychological interventions should be offered to them with priority. Moreover, psychological-related measures should be considered more important in daily preventive and therapeutic actions. It would be better to improve sleep facilities, optimize work arrangements, enrich psychological adjustment courses and so on, in order to improve resilience and ease the level of insomnia, work-family conflict and job burnout. As the influencing factors of both depression and anxiety and their co-occurrence in our study, healthier physical condition, insomnia and job burnout were the three factors that deserve the most attention when developing related prevention strategies in the post-pandemic era. Based on the above discussion, relevant authorities can further improve the mental health among healthcare workers in the post-pandemic era by offering regular physical check-ups and advice, alleviating insomnia, reducing work-family conflict and burnout, and improving psychological resilience. However, considering data on the risk factors of mental health problems among healthcare workers in the post-pandemic era remains lacking, much more studies are warranted to support the findings in our study.

Several limitations of this study should be noted. Firstly, data were collected through an online survey platform, which may lead to potential reporting bias due to social desirability. Secondly, all respondents of this study were healthcare workers at one hospital in Shenzhen, which may affect the representativeness of the samples, and whether the results of this study are applicable to healthcare workers in other regions during the post-pandemic era remains to be further studied. However, in the study period, majority of hospitals face with the similar situation for healthcare workers, where a series of strict prevention measures were taken to control the epidemic of COVID-19. Healthcare workers not only did the daily healthcare works, but also have to be involved in the COVID-19 prevention related works such as large-scale nucleic acid testing. Under the same country-level background of the post-pandemic era with routine prevention and control measures embedded, we think the study findings have a higher possibility to be applied to the similar hospital in other areas event outside Shenzhen. Besides, we sent out questionnaires to 400 recruited subjects, but only received 245 ones in our study, with a relatively low response rate of 61.25%. We cannot make a comparison of the demographic information between the respondents and non-respondents, because we conduct an anonymous investigation and we cannot identify the personal information of the non-respondents. However, similar studies of healthcare workers during the COVID-19 pandemic also had a response rate of around 60% (92, 93). This might depend on the nature of studied populations (94). According to the study of Rindfuss et al. (95), no evidence of bias was found from low response rates when examining the relationships in a multivariate analysis controlling for a variety of background variable, suggesting the reliability of our findings on the two hypotheses. Moreover, a cross-sectional design was conducted among healthcare workers at a tertiary hospital in Shenzhen in this study, so it is difficult to determine the direction or causality among the studied variables based on our data. In measuring mental health status at only one point, our study lacks relevant data about depressive and anxiety status and other variables before and during the pandemic, preventing us from comparing psychological symptoms interested in different periods. Finally, although depressive and anxiety symptoms are regarded as the main psychological problems on healthcare workers in our study, other psychological factors such as insomnia, job burnout and work-family conflict may also be influenced by the above two symptoms. The potential reverse effects among these variables remain to be further explored. If possible, the form of field questionnaire would allow us to obtain more reliable information in the process of collection and analysis in future. In the aspect of sampling method, we should formulate better sampling rules and certain prize mechanism, so as to improve the representativeness of samples and the response rate of questionnaires. A longitudinal study is required to evaluate the prevalence of depressive and anxiety symptoms, the ascertained directions of the associations and other potential psychological factors with the constant changes of COVID-19 during the post-pandemic era, and multi-center studies are needed to justify the findings of this study in the region.

## 5. Conclusion

To conclude, mental health problems were common among healthcare workers in Shenzhen during the post-pandemic era of COVID-19, with 34.7, 59.6, and 33.1% of healthcare workers reporting depressive and anxiety symptoms and their co-occurrence, respectively. We identified that job title, physical condition, insomnia, psychological resilience, work-family conflict and job burnout were still significant factors on healthcare workers' mental health, same as the case during the pandemic. Some factors such as job title during the pandemic may also play a role in the post-pandemic era, but with different effects. The findings suggest that healthcare authorities and policymakers should adopt targeted interventions, offer regular physical advice, provide professional psychological counseling, and strengthen communication among colleagues and leaders, thus improving mental health and reducing the appearance of psychological problems such as depression and anxiety symptoms in healthcare workers.

## Data availability statement

The raw data supporting the conclusions of this article will be made available by the authors, without undue reservation.

## Ethics statement

The studies involving human participants were reviewed and approved by the Ethics Committee of Shenzhen Qianhai Shekou Free Zone Hospital. The patients/participants provided their written informed consent to participate in this study.

## Author contributions

ZL wrote the first draft of the paper. YW and XW carried out the conception and design of the research. JM, JW, and SH participated in the acquisition of data. ZL and WW carried out most of the data collection. The work was critically revised by PQ. All authors commented on previous versions of the paper, as well as read, and approved the final version.
